# Functional and anatomical changes in diabetic macular edema after hemodialysis initiation: One-year follow-up multicenter study

**DOI:** 10.1038/s41598-020-64798-4

**Published:** 2020-05-08

**Authors:** Yoshihiro Takamura, Takehiro Matsumura, Kishiko Ohkoshi, Tatsuhiko Takei, Kunihiro Ishikawa, Masahiko Shimura, Tetsuo Ueda, Masahiko Sugimoto, Takao Hirano, Kei Takayama, Makoto Gozawa, Yutaka Yamada, Masakazu Morioka, Masayuki Iwano, Masaru Inatani

**Affiliations:** 1 J-CREST (Japan Clinical REtina STudy group), Kagoshima, Japan; 2 Department of Ophthalmology, Faculty of Medical Sciences, University of Fukui, Yoshida, Fukui Japan; 3 Department of Ophthalmology, St. Luke’s International Hospital, Tokyo, Japan; 4 Department of Ophthalmology, Diabetes Center, Tokyo Women’s Medical University, Tokyo, Japan; 5 Department of Ophthalmology, Tokyo Medical University Hachioji Medical Center, Hachioji, Tokyo Japan; 6 Department of Ophthalmology, Nara Medical University Graduate School of Medicine, Kashihara, Nara Japan; 7 Department of Ophthalmology, Mie University Graduate School of Medicine, Tsu, Mie Japan; 8 Department of Ophthalmology, Shinshu University School of Medicine, Matsumoto, Nagano Japan; 9 Department of Ophthalmology, National Defense Medical College, Tokorozawa, Saitama Japan; 10 Department of nephrology, Faculty of Medical Sciences, University of Fukui, Yoshida, Fukui Japan

**Keywords:** Medical research, Nephrology

## Abstract

Diabetic nephropathy and retinopathy (DR) including diabetic macular edema (DME) are representative microvascular complications of diabetes. We conducted a retrospective multicenter study analyzing records from patients with DR (132 eyes in 70 patients) and end-stage renal diseases (ESRD) who underwent hemodialysis for the first time. We demonstrated that the central retinal thickness (CRT) values were significantly decreased (p < 0.0001), and the best-corrected visual acuity (BCVA) values were improved (p < 0.05) at 1, 3, 6, 9, and 12 months after hemodialysis initiation, in spite of a lack of specific ocular treatments for DME in 93.2% of eyes. We found a significant positive correlation in the rates of CRT changes between right and left eyes. The CRT reductions were greater in eyes with DME type subretinal detachment than in those with spongelike swelling and cystoid macular edema. The visual outcome gain was associated with the CRT reduction at 12 months in the eyes with good initial BCVA (≧20/50). Hemodialysis induction contributed to functional and anatomical improvements after 1 year, independently of initial laboratory values before the hemodialysis.

## Introduction

Diabetes mellitus (DM) is a chronic disease characterized by persistent hyperglycemia requiring continuous care and management. Diabetic retinopathy (DR) and nephropathy (DN) are representative microvascular complications of DM^1^. DR and DN are life-threatening because these complications can lead to blindness and end-stage renal diseases (ESRD), respectively^1^.

In patients with DR, the visual function can be severely damaged by complications of diabetic macular edema (DME)^2^. DME results from the hyperpermeability of retinal vessels, and intravitreal injection of anti-vascular endothelial growth factor (VEGF) agents has become a gold standard in DME treatment^3–5^. However, many patients with DME show a low response to anti-VEGF treatment, and repeated injections are required to maintain its therapeutic effects^6–8^.

Dialysis is an effective treatment modality for ESRD. In Japan, the number of patients undergoing dialysis increases yearly, and the prevalence was 2,597 patients per million population in 2016^9^. DN was the most common kidney disease (38.8%) requiring dialysis in 2016, followed by chronic glomerulonephritis (28.8%) and nephrosclerosis (9.9%). ESRD and DME are sometimes present in the same patient, and assessing the effect of hemodialysis on DME is clinically important.

Whether dialysis influences the status of DME remains controversial^10–14^. Tokuyama *et al*. found that angiography fluorescein leakage remained constant after dialysis^10^. On the other hand, Perkovich *et al*. reported that hemodialysis was involved in the resolution of hard exudates and DME^11^. In contrast to these subjective estimations, an objective and quantitative method using optical coherence tomography (OCT) may be more accurate at estimating subtle changes in the retinal thickness. A study found significant reductions in macular thickness by OCT immediately after dialysis, but another one found no changes 30 minutes after hemodialysis^12,13^. However, in these studies, the enrolled patients were already on dialysis, and thus, the effect of the hemodialysis initiation is unclear. To clarify this issue, Hwang *et al*. showed that central retinal thickness (CRT) decreased significantly at 1 month after the first hemodialysis^14^. These studies were small scaled and had short observation terms, and no studies have assessed the DME effects of hemodialysis initiation for longer periods. Therefore, we conducted a large-scale, retrospective, multicenter study and assessed the temporal profiles of CRT and best-corrected visual acuities (BCVAs) for 1 year in patients with DM and ESRD after hemodialysis initiation.

## Results

### Anatomical change of DME after initiation of hemodialysis

In all, we enrolled 132 eyes from 70 patients in this study. In eight patients, we only included one eye in the analysis, because the other eight eyes did not meet the inclusion criteria: five eyes from five patients had severe corneal opacities with neovascular glaucoma and three had a tractional retinal detachment. Table 1 lists characteristics of the patients at baseline. The mean age was 58.91 ± 10.37 years. Among 132 eyes, 36, 49, and 47 were mild non-proliferative DRs, severe non-proliferative DRs, and proliferative DRs, respectively. We found 118 eyes (89.4%) with DME before the first hemodialysis.

**Table 1:** Patients’ characteristics.

Variables	Baseline (SD)
Number of patients (eyes)	70 (132)
Age (years)	58.91 ± 10.37
Right/left	68/64
Gender (men/women)	46/24
Duration of diabetes mellitus (years)	16.18 ± 9.03
Height (cm)	165.03 ± 10.26
Body weight (kg)	70.97 ± 16.38
Systolic BP (mmHg)	155.40 ± 26.20
Diastolic BP (mmHg)	80.31 ± 16.36
Hemoglobin A1c (%)	8.52 ± 1.01
BUN (mg/dL)	75.82 ± 25.75
Creatinine (mg/dL)	8.71 ± 3.13
eGFR (mL/min/1.73m^2^)	6.08 ± 2.76
LDL (mg/dL)	92.79 ± 38.56
HDL (mg/dL)	49.43 ± 17.49
TG (mg/dL)	143.72 ± 74.76

BUN: blood urea nitrogen, eGFR: estimated glomerular filtration rate, BP: Blood pressure, LDL: low density lipoprotein, HDL: high density lipoprotein, TG: triglyceride.

**Figure 1:**
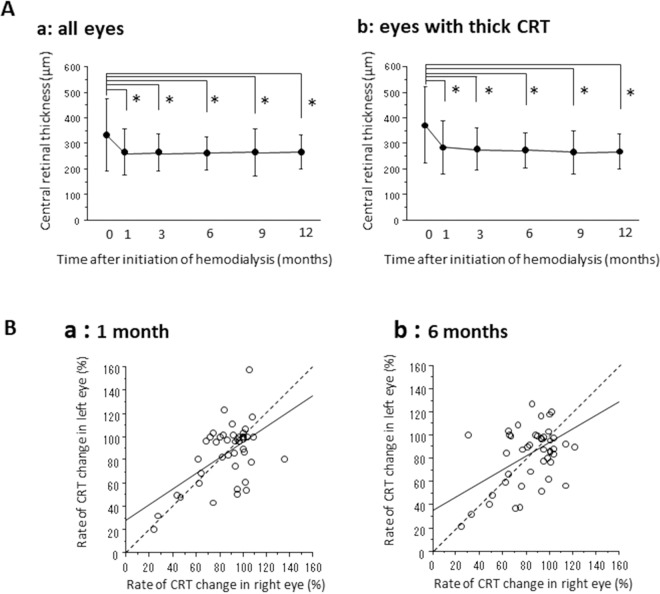
OCT findings after hemodialysis initiation. (**A**) Changes in CRT after hemodialysis initiation in all eyes (a) and in the eyes with thick CRTs at baseline (b). Data represent means ± standard deviations (SD). *p < 0.05 (versus baseline). (**B**) Linear correlation in the rate of CRT changes between the right eye and the left eye. We found significant association at 1 (**A**; P < 0.0001, R^2^ = 0.323), and 6 (**C**; P = 0.002, R^2^ = 0.202). Proximity of the point to the broken line implies a similar CRT rate change between right and left eyes.

Figure 1A shows the temporal CRT profiles before and after starting hemodialysis in all eyes (a) and in one side of eyes showing thick CRTs at baseline (b). In all eyes, the mean CRTs decreased significantly to 273.4 ± 95.3 μm at 1 month, to 273.7 ± 101.9 μm at 3 months, to 261.8 ± 69.7 μm at 6 months, to 261.3 ± 89.1 μm at 9 months, and to 266.8 ± 78.5 μm at 12 months compared to the mean baseline at 334.0 ± 142.6 μm (p < 0.0001 for each time point). The data of the eyes with thick CRTs also showed a significant decrease from 373.2 ± 149.5 μm to 285.5 ± 102.5 μm at 1 month, to 279.7 ± 81.7 μm at 3 months, to 275.9 ± 69.2 μm at 6 months, to 266.1 ± 84.9 μm at 9 months, and to 268.5 ± 68.7 μm at 12 months (p < 0.0001 for each time point). We investigated the association between the CRT change rates in right and left eyes (Fig. 1B) and found significant positive correlations at 1 month (p < 0.0001, R^2^ = 0.323), 3 months (p < 0.0001, R^2^ = 0.323), 6 months (p = 0.002, R^2^ = 0.202), and 12 months (p = 0.0008, R^2^ = 0.269). The numbers of eyes with intravitreal anti-VEGF injection and sub-Tenon’s triamcinolone acetonide (STTA) injection were seven eyes (5.3%) (5 eyes: aflibercept, 2 eyes: ranibizumab) and two eyes (1.5%), respectively, during 12 months after hemodialysis initiation. The other 123 eyes (93.2%) had no injections of anti-VEGF agents or steroids during the observational periods.

In 24.2% of eyes with DME (32/132 eyes), there was a history of intravitreal injections of anti-VEGF drugs and STTA for 1 year prior to the first hemodialysis. In these 32 eyes, the CRT significantly decreased from 349.5 ± 128.4 μm at baseline to 287.5 ± 83.4 μm at 1 month after initiation of hemodialysis (p < 0.0001). No additional injections were performed in 78.1% (25/32 eyes) after first hemodialysis. The average numbers of injections were 2.96 ± 1.92 and 0.25 ± 0.56 for 1 year before and after initiation of hemodialysis, respectively.

### Subretinal detachment was sensitive type of DME to induction of hemodialysis

**Figure 2:**
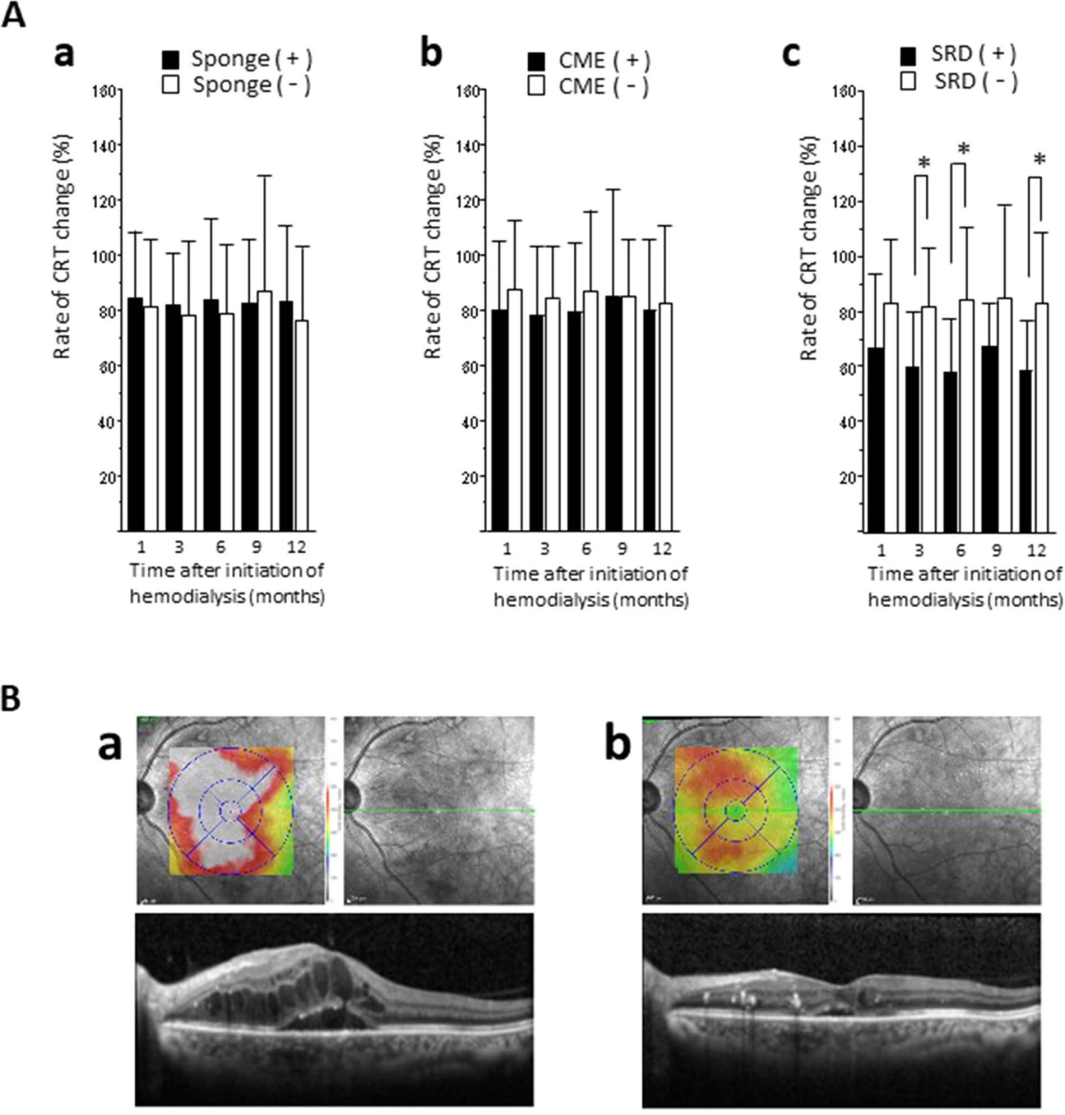
OCT findings after hemodialysis initiation according to different types of DME. (**A**) CRT change rates between the presence (black bar) and absence (white bar) of spongelike swelling (a), CME (b), and SRD (c). We found significant differences (*p < 0.05) in SRD type at 3, 6, and 12 months after hemodialysis initiation. (**B**) Representative case showing OCT changes after hemodialysis initiation. A 46-year-old man with ESRD and DME initiated hemodialysis. Previous OCTs showed severe SRD (a). At 1 month after the hemodialysis initiation, DME had improved (b), and the BCVA improved from 20/100 to 20/40. Cross-sectional image corresponds to the green line.

Next, we investigated the type of DME that was sensitive to the induction of hemodialysis. We classified 118 eyes with DME into 3 types including those with spongelike swelling, cystoid macular edema (CME), and subretinal detachment (SRD). At baseline, the incidences for eyes with spongelike swelling, CME, and SRD were 52.5% (62/118), 49.2% (58/118), and 20.3% (24/118), respectively (the number was higher than 118 because some DME types were present on the same eye). We compared the CRT change rates after hemodialysis initiation between the eyes with and without spongelike swelling, CME, or SRD (Fig. 2A). We found no significant differences in the eyes with the spongelike swelling and CME, but the rate of CRT change in the eyes presenting SRD was significantly lower than that in eyes without SRD at 3 months (p = 0.0304), 6 months (p = 0.0135), and 12 months (p = 0.0305) after hemodialysis initiation. Figure 2B shows the OCT findings of a representative eye with SRD. Before hemodialysis initiation, we observed severe macular swelling on the OCT map and cross-sectional images. One month after dialysis initiation, the CRT decreased from 603 μm to 293 μm, and the BCVA improved from 20/100 to 20/40. Cross-sectional OCT images showed that the subretinal fluid space disappeared in 25.0% (6/24), 16.7% (4/24), and 58.3% (14/24) of the eyes with SRD at 1 month, 3 months, and 6 months, and we did not detect any subretinal fluid at either 9 or 12 months.

### BCVA changes after hemodialysis initiation

**Figure 3:**
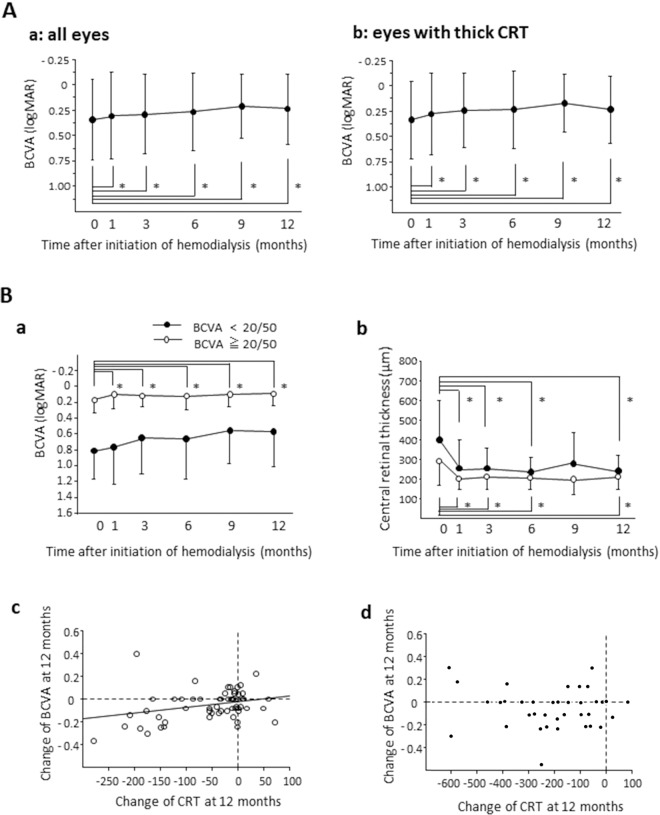
BCVA change after hemodialysis initiation and relation to CRT. (**A**) BCVA changes after hemodialysis initiation in all eyes (a) and in the eyes with thick CRTs at baseline (b). Visual acuity is expressed as the logMAR. Data represent means ± standard deviations (SD). *p < 0.05 (versus baseline). (**B**) Temporal profiles of BCVA and CRT in the patients with good BCVA (≧20/50) and poor BCVA (<20/50) at baseline. Changes of BCVA (a) and CRT (b) after hemodialysis initiation in the patients with good BCVAs (white circle) and poor BCVAs (black circle). *p < 0.05 (versus baseline). We found a significant correlation between changes of BCVA and CRT at 12 months after hemodialysis initiation in the patients with good BCVAs (c: p = 0.028, R^2^ = 0.084), but not in the patients with poor BCVAs (d). The broken line indicates the baseline CRT and BCVA values.

Figure 3A shows the BCVA changes after hemodialysis initiation in all eyes (a) and the eyes with thick CRTs at baseline (b). In all eyes, the mean BCVAs improved significantly from 0.353 ± 0.365 to 0.318 ± 0.426 at 1 month (p = 0.0011), to 0.297 ± 0.386 at 3 months (p = 0.0012), to 0.276 ± 0.382 at 6 months (p = 0.0023), to 0.239 ± 0.326 at 9 months (p = 0.0028), and to 0.258 ± 0.361 at 12 months (p = 0.0030). In the eyes with thick CRTs, the mean BCVA also improved from 0.335 ± 0.383 to 0.276 ± 0.405 at 1 month (p = 0.0026), to 0.245 ± 0.369 at 3 months (p = 0.0018), to 0.234 ± 0.386 at 6 months (p = 0.0135), to 0.173 ± 0.279 at 9 months (p = 0.0003), and to 0.234 ± 0.329 at 12 months (p = 0.0265).

Based on the initial BCVAs, we separated eyes into two groups, eyes with good BCVA (20/50 and higher) and eyes with poor BCVA (lower than 20/50), and then analyzed the BCVA changes (Fig. 3Ba). In the eyes with good BCVA, the BCVA improved significantly from 0.160 ± 0.198 to 0.111 ± 0.188 at 1 month (p < 0.0001), to 0.103 ± 0.167 at 3 months (p < 0.0001), to 0.115 ± 0.187 at 6 months (p = 0.027), to 0.0994 ± 0.167 at 9 months (p = 0.0017), and to 0.093 ± 0.173 at 12 months (p = 0.0038). On the other hand, the eyes with poor initial BCVA showed no significant BCVA improvements through the observational periods. We also analyzed the CRT changes after hemodialysis initiation (Fig. 3Bb). In the eyes with good BCVA, we found significant CRT decreases at 1, 3, 6, and 12 months (p < 0.0001 for each time point). In the eyes with poor BCVA, we found significant CRT decreases at 1 (p < 0.0001), 3 (p = 0.0001), 6 (p = 0.0006), and 12 months (p = 0.0027). We found a significant correlation between the BCVA change and the CRT at 12 months in the eyes with good initial BCVA (p = 0.028, R^2^ = 0.084) (Fig. 3Bc) but found no such association in the eyes with poor initial BCVA (Fig. 3Bd).

We found no significant correlation between renal function markers including serum creatinine (Cr), blood urea nitrogen (BUN), and estimated glomerular filtration rate (eGFR), or systemic factors such as hemoglobin A1c (HbA1c), high-density lipoprotein (HDL), low-density lipoprotein (LDL), triglycerides (TG), body weight, height, systolic BP, diastolic BP before the hemodialysis initiation and BCVA or CRT change at any time point.

## Discussion

The data of this retrospective multicenter study showed that DME and BCVA improved significantly after the induction of hemodialysis in the eyes with DR of patients with ESRD. The significant CRT reductions were induced rapidly at 1 month after hemodialysis initiation. This finding is consistent with the results of a small-scale study by Hwang *et al*.^14^ showing the CRT change 1 month after hemodialysis initiation. In our study, we showed that the CRT reduction effects were maintained at least for 1 year. We analyzed data from patients who had started hemodialysis for the first time and thus were able to estimate the exact effect on the DME and visual acuity. Also, the accumulation of data from multiple centers enabled us to analyze a large number of cases. To avoid potential biases, we analyzed the data of one eye from each patient, as well as data from both eyes from each patient, and obtained similar results. The enrollment of both eyes led us to find a significant correlation between the CRT changes in the right and left eyes. This indicates that the CRT of the right and left eyes changed in parallel after the hemodialysis initiation. It is possible that the normalization of systemic conditions by the dialysis affected both eyes. Our findings also suggest that renal function systemic factors are associated with the pathogenesis of DME. We observed significantly positive correlations at 1, 3, 6, and 12 months that the hemodialysis effects on the eyes were long-lasting.

Intravitreal anti-VEGF injections have become a standard DME treatment^3–5,15,16^. Since most eyes in our study (94.7%) had no intravitreal anti-VEGF injections during the year after hemodialysis initiation, we unlikely believe that anti-VEGF therapy contributed to the CRT reductions we observed. The small number of anti-VEGF injections in our patients may be due to the rapid CRT decreases after induction of the hemodialysis, making additional treatments unnecessary. Also, given the poor systemic conditions of patients in this study, the clinicians may have moderated the use of medications to avoid adverse effects of the drugs on the whole body^16^. Eyes with the history of anti-VEGF therapy for DME also showed the significant reduction of CRT and decrease in the number of injections after the initiation of hemodialysis. Based on this result, it is possible that the induction of the hemodialysis may be effective to treat the refractory DME cases to anti-VEGF therapy.

Our data demonstrates that eyes with DME and SRD showed a greater CRT reduction than those without SRD. This suggests that the DME classification, based on OCT images, may have important clinical implications for predicting anatomical improvement of DME in response to hemodialysis induction. SRD is common in DME with a reported incidence of 13% to 45%^17,18^ that reached 20.3% in our case series. Cross-sectional OCT images of all DME cases with SRD in our study showed that the area of subretinal fluid had disappeared at 6 months after hemodialysis initiation. The mechanism by which hemodialysis initiation may have promoted the absorption of the subretinal fluid is unclear, but we believe that the mutual flow of the excess fluid between the retina and the choroidal tissue or the retinal pigment epithelium may have improved after hemodialysis. Other studies have shown significant decreases of choroidal thickness after hemodialysis^14,19–21^. Ishibazawa *et al*. showed that the reduction of choroidal thickness was greater in patients with diabetes than in patients without it after hemodialysis, implying that systemic fluid accumulation has a greater effect on the diabetic choroid, probably due to damage to the choroidal vasculature, in patients with ESRD^19^. The significant increase in vessel diameter after hemodialysis reflects the ease by which it changes the retinal circulation^22^. The dynamic changes in retinal and choroidal circulations may be associated with the DME improvement. Based on our data, the CRT reduction rates in the eyes with spongelike swelling and CME were approximately 20%, while that of the eyes with SRD was 40%. These data suggest that the SRD type is particularly responsive, although our results showed edema improvements also for the eyes with spongelike swelling and CME after hemodialysis.

We demonstrated that the BCVAs also improved significantly at 1 month and thereafter after hemodialysis initiation. Introducing hemodialysis may contribute to functional and anatomical improvements in patients with ESRD. Moreover, our subgroup analysis showed significant BCVA improvements in the patients with better initial BCVAs (≧20/50), but not in the patients with poor initial BCVAs (less than 20/50). In the group with good initial BCVA, we found a significant association between the change of BCVA and the CRT at 12 months, and thus, the recovery from edema may have resulted in the improved visual outcomes. On the other hand, the group with poor initial BCVA had no significant visual recovery despite a good anatomical response, and we did not find a significant correlation between the CRT change and the BCVA at 12 months. Therefore, the absorption of excess fluid in retinal tissues did not lead to vision recovery in the eyes with poor initial BCVA. Different causes of functional impairment have been proposed including ischemia, glial reactivity, apoptosis, and photoreceptor integrity^23–25^. In the eyes with these impairments, visual disturbances may be irreversible, even after edema resolution by hemodialysis. Based on our data, the visual acuity at baseline may be clinical predictor of the visual outcome after hemodialysis initiation.

In this study, we found no significant association between the BCVA or CRT changes and systemic values at baseline including those for serum Cr, BUN, eGFR, lipids, BP, or HbA1c. The renal and systemic statuses, which were extremely poor in the patients scheduled to undergo hemodialysis, improved dramatically and rapidly after the first dialysis. Even with some variation in the laboratory values, we expect that improvements in the systemic condition after hemodialysis may effectively improve the visual acuity of patients.

This study had some limitations. Due to its multicenter nature, the conditions determining the hemodialysis initiation timing varied according to each facility. However, we confirmed the significance in the improvements of CRT and BCVA by analyses in each facility (data not shown), strengthening the reliability of our findings. Also, we did not collect blood samples after hemodialysis at the same times; thus, we could not investigate the laboratory value changes after hemodialysis and their associations to the CRT and BCVA changes. Further prospective studies are required to clarify this issue.

In conclusion, our multicenter study demonstrated that hemodialysis initiation results in improvements in DME (especially in those of the SRD type) and in BCVA in patients with ESRD. The CRT changes in right and left eyes were parallel, implying an association between DME pathogenesis and the systemic conditions modified by hemodialysis. Better visual acuity at baseline may be important for obtaining the best visual outcomes after dialysis. Determining the appropriate hemodialysis initiation timing is difficult, but our data suggests that the therapeutic effect on the DME may provide a basis for recommending that patients with ESRD and DME undergo hemodialysis.

## Methods

We collected data from eight clinical centers throughout Japan. The current study was performed in accordance with the Declaration of Helsinki and with approval from the University of Fukui Institutional Review Board and the ethics committees of the other participating hospitals. All patients provided signed informed consent forms. We registered the study with the University Hospital Medical Information Network Clinical Trials Registry (UMIN-CTR) of Japan (ID UMIN 000038081; date of access and registration, September 23, 2019). We retrospectively reviewed the medical records of patients with DR, who started hemodialysis due to ESRD for the first time. The primary objective of this study is to identify any CRT and BCVA changes after dialysis initiation. The secondary objective was to find associations between the types of DME (including spongelike swelling, CME, and SRD) and the hemodialysis induction DME changes. The third objective was to assess any correlations between BCVA changes and the laboratory values before the hemodialysis initiation including serum levels of BUN, blood sugar, HbA1c, Cr, eGFR, total protein, TG, LDL, and HDL.

We enrolled patients with ESRD due to type 2 DM who had started hemodialysis for the first time and who had had it for at least 12 months in each medical center. We invited patients with DR, 20 years and older, who were regularly treated in the hemodialysis unit 3 times a week to participate in this study. We defined the grade of DR in accordance with the criteria of the International Clinical Disease Severity Scale for DR^26^. We excluded patients 1) with retinal diseases other than DR; 2) with severe cataract, corneal diseases, or vitreous hemorrhage resulting in poor-quality OCT image; 3) with intravitreal injection of anti-VEGF drugs and STTA within 3 months before the dialysis start; and 4) treated by peritoneal dialysis.

All patients underwent a complete ophthalmic examination, including BCVA, fundus examination, and OCT (SPECTRALIS, Heidelberg Engineering, Vista, CA, USA) before and at 1, 3, 6, 9, and 12 months after the initiation of the hemodialysis. CRTs were automatically measured as the average retinal thicknesses within a 500 μm radius using the macular thickness protocol. We calculated the CRT rate changes by dividing the CRT before initiation of dialysis by the last measurement. We converted the BCVAs to the logarithm of the minimum angle of resolution (logMAR) scale. We analyzed the temporal profiles of CRT and BCVA in both eyes and one side of eyes in which CRT was greater than other eyes at baseline and defined as “eyes with thick CRT”. We measured eGFR, Cr, BUN, HbA1c, serum albumin, and lipids (HDL, LDL, and TG) in blood samples before the hemodialysis. We reviewed body weight, height, and systolic and diastolic BPs using the patients’ medical records. We classified DME based on OCT scans into three types including spongelike swelling, CME, and SRD^27^. We included eyes with images showing both CME and SRD into both CME and SRD groups.

We performed statistical analyses using JMP (SAS institute, Tokyo, Japan). We compared CRTs and BCVAs among the different time points using the Wilcoxon signed-rank test and considered differences with p-values <0.05 as statistically significant. We expressed data as means ± standard deviations (SD). We used ordinary least square regression analyses to assess correlations between BCVA or CRT and laboratory values before the initiation of dialysis and between the rates of CRT changes of right and left eyes, and CRT changes and BCVA changes at 12 months.

## Data Availability

The datasets generated during and/or analyzed during the current study are available from the corresponding author on reasonable request.
